# Molecular Mechanisms in Amyotrophic Lateral Sclerosis: The Role of Angiogenin, a Secreted RNase

**DOI:** 10.3389/fnins.2012.00167

**Published:** 2012-11-19

**Authors:** Isabela M. Aparicio-Erriu, Jochen H. M. Prehn

**Affiliations:** ^1^Department of Physiology and Medical Physics, Centre for the Study of Neurological Disorders, Royal College of Surgeons in IrelandDublin, Ireland

**Keywords:** amyotrophic lateral sclerosis, RNA metabolism, angiogenin, RNA binding proteins, stress signals

## Abstract

Amyotrophic lateral sclerosis is a fatal neurodegenerative disease caused by the loss of motoneurons. The precise molecular and cellular basis for neuronal death is not yet well established, but the contemporary view is that it is a culmination of multiple aberrant biological processes. Among the proposed mechanisms of motoneuron degeneration, alterations in the homeostasis of RNA binding proteins (RBP) and the consequent changes in RNA metabolism have received attention recently. The ribonuclease, angiogenin was one of the first RBPs associated with familial and sporadic ALS. It is enriched in motoneurons under physiological conditions, and is required for motoneuron survival under stress conditions. Furthermore, delivery of angiogenin protects cultured motoneurons against stress-induced injury, and significantly increases the survival of motoneurons in SOD^G93A^ mice. In this overview on the role of angiogenin in RNA metabolism and in the control of motoneuron survival, we discuss potential pathogenic mechanisms of angiogenin dysfunction relevant to ALS and other neurodegenerative disorders. We also discuss recent evidence demonstrating that angiogenin secreted from stressed motoneurons may alter RNA metabolism in astrocytes.

## Introduction

Amyotrophic lateral sclerosis (ALS) is fatal neurodegenerative disease with a late-onset, where motoneurons in the spinal cord and brainstem die. After diagnosis, only about 25% of patients survive beyond 5 years, with the majority suffering a fatal respiratory failure within 3–5 years. Most cases are believed to be sporadic, with only about 10% of patients having a confirmed family history.

Several genetic alterations have been linked with ALS. Mutations in the copper/zinc superoxide dismutase 1 (SOD1) gene, responsible for *circa* 20% of the familial ALS forms and 1% of “sporadic” cases, have been considered the major genetic cause of ALS (Rosen et al., 1993). Recently, however, an expanded non-coding GGGGCC repeat in *C9ORF*72 has been identified, which seems to be responsible for about 24% of familial ALS (DeJesus-Hernandez et al., 2011). In a Finnish cohort, the percentage of linkage for this mutation was as high as 46% of ALS, putting this as the most common genetic cause of ALS known to date (Renton et al., 2011). Other important genes linked to ALS include two RNA binding proteins, transactive response (TAR) DNA-binding protein (TDP-43), and fused in sarcoma/translocated in liposarcoma (FUS/TLS), which are associated with *circa* 4% of familial ALS (reviewed by Lagier-Tourenne et al., 2010).

Still, the large majority of sporadic cases have no known genetic component (reviewed by Valdmanis and Rouleau, 2008). These observations have led to the hypothesis of ALS being an oligogenic or polygenic disorder, a hypothesis that could also explain the large number of familial ALS-associated gene mutations that exhibit a relatively low penetrance (Valdmanis and Rouleau, 2008).

## Angiogenin in ALS

A clinical study initiated in Ireland has identified several mutations in the angiogenin (*ANG*) gene in ALS patients of Irish and Scottish background, both in familial and sporadic cases (Greenway et al., 2006). Subsequent clinical studies confirmed the association of these mutations with ALS, and identified new mutations in backgrounds from Brazil, China, France, Germany, Italy, Netherlands, Sweden, and USA (Table 1). Only one clinical study so far failed to find a link between *ANG* gene mutations and ALS in an Italian population (Corrado et al., 2007), but one can arguably reason that such study was small (262 ALS patients) in comparison with other reports (with an average of approximately 1,500 ALS patients per study), and that some of the *ANG* mutations identified may have a lower disease penetrance, similar to other ALS-associated mutations (Valdmanis and Rouleau, 2008). More recently, a link between angiogenin mutations and Parkinson’s disease has also been demonstrated (Steidinger et al., 2011; van Es et al., 2011).

**Table 1:** **Angiogenin mutations associated with ALS**.

Mutation	Origin of disease	Ethnicity	Possible/knowneffect on function	Oligogenic model	Association with other neurodegenerative conditions
M(−24)S (Wu et al., 2007; Gellera et al., 2008)	Sporadic	Europe /America	Affect correct translation		
M(−24)I (van Es et al., 2011)	Sporadic	Europe	Affect correct translation		Parkinson’s disease (van Es et al., 2011)
F(−13)L (Fernández-Santiago et al., 2009; van Es et al., 2011)	Sporadic	Europe	Affect processing/traffic		
F(−13)S (Gellera et al., 2008; van Es et al., 2011)	Familial	Europe	Affect processing/traffic		
G(−10)D (van Es et al., 2011)	Sporadic	Europe	Affect protein function		
P(−4)Q	Sporadic	Europe	Affect processing/traffic		
P(−4)S (Wu et al., 2007; van Es et al., 2011)	Sporadic	America	Affect processing/traffic		Parkinson’s disease (van Es et al., 2011)
**Q12L** (Greenway et al., 2006; van Es et al., 2011)	**Sporadic**	**Europe**	**Loss of activity**		
**K17I** (Greenway et al., 2006; Wu et al., 2007; Millecamps et al., 2010; van Es et al., 2011; van Blitterswijk et al., 2012)	**Sporadic/ familial**	**Europe/ America**	**Loss of activity**	**TDP-43 FUS/TLS**	**Frontotemporal dementia** (van Es et al., 2009)
**K17E** (Greenway et al., 2006; van Es et al., 2009; van Es et al., 2011)	**Sporadic**	**Europe**	**Loss of activity**		
S28N (Wu et al., 2007; van Es et al., 2011)	Sporadic	America	Impaired nuclear translocation/loss of activity		
**R31K** (Greenway et al., 2006; van Es et al., 2011)	**Sporadic**	**Europe**	**Impaired nuclear translocation**		
**C39W** (Greenway et al., 2006; van Es et al., 2011)	**Familial**	**Europe**	**Loss of activity**		
**K40I** (Greenway et al., 2006; van Es et al., 2011)	**Sporadic**	**Europe**	**Loss of activity**		
**I46V** (Greenway et al., 2006; Gellera et al., 2008; Conforti et al., 2008; Paubel et al., 2008; Fernández-Santiago et al., 2009; van Es et al., 2011)	**Familial/ sporadic**	**Europe**	**Loss of activity**		
K54E (Fernández-Santiago et al., 2009; Millecamps et al., 2010; van Es et al., 2011)	Sporadic/ familial	Europe	Affect interaction with nucleic acids/proteins	FUS/TLS	
T80S (van Es et al., 2011)	Sporadic	Europe	Tolerated/affect protein function		
F100I (van Es et al., 2011)	Sporadic	Europe	Tolerate/benign		
V103I (Zou et al., 2012)	Sporadic	Asia	n.a.		
P112L (Wu et al., 2007; van Es et al., 2011)	Sporadic	America	Impaired nuclear translocation/loss of activity		
V113I (Gellera et al., 2008; van Es et al., 2011)	Sporadic/ familial	Europe	Tolerated/affect protein function		
H114R (Gellera et al., 2008; van Es et al., 2011)	Familial	Europe	Loss of activity		
R121H (Paubel et al., 2008; Millecamps et al., 2010; van Es et al., 2011)	Sporadic/ familial	Europe	Loss of activity		
R145C (van Es et al., 2011; Luigetti et al. 2011)	Sporadic	Europe	n.a.	SOD1	
g.446C→T (Gellera et al., 2008; UTR region)	Sporadic	Europe	Affect gene expression		

*n.a., not available/analyzed; Bold, These mutations have been biochemically characterized by Crabtree et al., 2007*.

Angiogenin, firstly isolated from the conditioned medium of colon carcinoma cells (Fett et al., 1985), is a member of the pancreatic RNase A superfamily, recently renamed as vertebrate secreted RNases (Li and Hu, 2012). This RNase is characterized by an unusual low catalytic activity, but has a significant biological ability to induce angiogenesis – hence its name (reviewed by Tello-Montoliu et al., 2006; Table 2). Most of the ALS-linked *ANG* mutations are predicted to affect the catalytic activity or cellular localization of the enzyme (summarized in Table 1), suggesting that the associated phenotype in ALS is mainly caused by loss of activity. The mutants identified by Greenway and colleagues were biochemically characterized (Crabtree et al., 2007), and all but the *ANG^R31K^* mutant showed a marked reduction in catalytic activity as predicted from structural studies.

**Table 2:** **Functions associated with angiogenin**.

Function	Mechanism	RNAse activity-dependent	Reference
Angiogenesis/wound healing	Activation of PLC signal pathway	Yes, depends on nuclear translocation	Fett et al. (1985); Bicknell and Vallee (1988); Moroianu and Riordan (1994); Liu et al. (2001); Pan et al. (2012)
	Activation of Erk1/2 signal pathway		
	rRNA synthesis		
Neurite growth and pathfinding	Unclear	Yes – angiogenin inhibitor blocks function	Subramanian and Feng (2007); Subramanian et al. (2008)
Neuroprotection	Activation of PI3K/Akt signal pathway	Yes – loss of protection with inactive ALS-associated mutants	Kieran et al. (2008); Sebastia et al. (2009); Steidinger et al. (2011); Skorupa et al. (2012)
	Engagement of HIF-1α		
	Paracrine signaling	
Response to stress	Inhibition of protein translation	Yes – cleavage of rRNA	Emara et al. (2010); Fu et al. (2009); Yamasaki et al. (2009); Ivanov et al. (2011)
	Assembly of stress granules	Yes – cleavage of tRNA	

*PLC, Phospholipase C; PI3K, Phosphatidylinositol 3-kinase*.

Angiogenin has long been associated with different pathological conditions, such as cancer and angiogenesis, neovascularization associated with diabetic retinopathy and ischemia, as well as rheumatoid arthritis (reviewed by Adams and Subramanian, 1999). Early studies with endothelial cells have identified angiogenin as a hypoxia-inducible, secreted protein which acts as a potent inducer of rRNA transcription and neovascularization (Moroianu and Riordan, 1994). The potential involvement of angiogenin as a neuronal signaling molecule relevant to ALS therefore came as a surprise. However a previous study has associated another hypoxia-inducible, angiogenic, and neurotrophic factor, VEGF, to play a role in ALS (Lambrechts et al., 2003).

**Figure 1:**
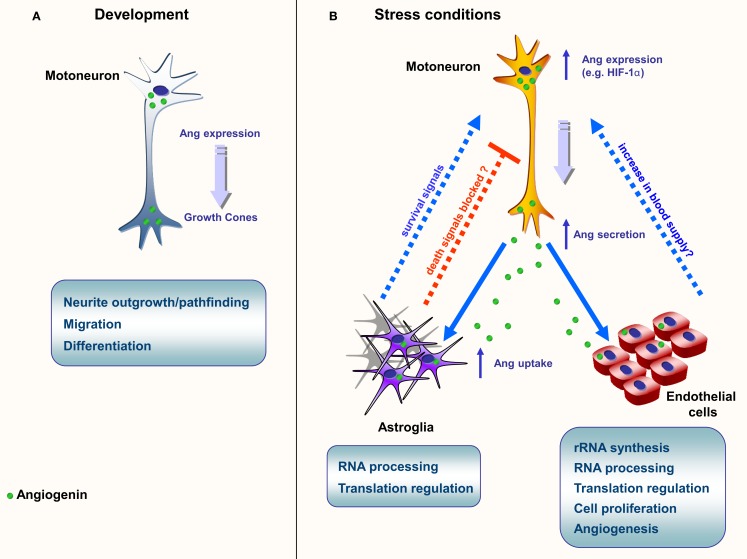
**Schematic representation of the main known functions of angiogenin**. **(A)** Neurite growth and pathfinding. Angiogenin is found in high levels during embryogenesis, both on the brain and spinal cord; **(B)** Neuroprotection in ALS models. In situations of stress, such as starvation and hypoxia, angiogenin expression is up-regulated in motoneurons. Angiogenin is secreted and endocytosed by surrounding astroglia and close endothelial cells. In astroglia, angiogenin processes RNA, possibly altering the protein translation profile. A similar phenomenon is proposed to happen in endothelial cells, culminating in the production of survival signals (astroglia) and angiogenesis (endothelial cells), possibly resulting in increase of blood flow in affected areas.

Angiogenin is expressed at high levels in the developing nervous system both in the brain and spinal cord, predominantly in neurons, and its activity has been shown to be necessary for neurite extension/pathfinding in differentiated motoneuron-like cells derived from pluripotent P19 carcinoma cells (Subramanian and Feng, 2007; Figure 1A and Table 2). Interestingly, ALS-associated angiogenin mutants failed to show the same activity (Subramanian et al., 2008). Angiogenin is also expressed and enriched in adult motoneurons (Greenway et al., 2006), and has been shown to protect mature, cultured motoneurons against different ALS-associated insults, such as excitotoxicity (Ca^2+^ mediated injury resulting from glutamate receptor overactivation), hypoxia, and endoplasmic reticulum stress. Angiogenin has been shown to promote and sustain cell survival signaling through AKT and ERK kinase pathways (Kieran et al., 2008; Sebastia et al., 2009). In addition, angiogenin delivery significantly increased the life-span and improved motor function in SOD1^G93A^ mice, an established mouse model of ALS when delivered post-symptom onset (Kieran et al., 2008).

## Paracrine Activity of Angiogenin

Recently our group has provided compelling evidence of a new signaling pathway between motoneurons and astroglia mediated by angiogenin (Skorupa et al., 2012). Our data indicate that angiogenin is a neuronally produced protein which may constitutively regulate RNA cleavage in motoneurons (Figure 1B). However both transcription and secretion of angiogenin by motoneurons is potently activated in response to stress, and motoneuron-derived, secreted angiogenin is subsequently taken up nearly exclusively by astroglia (Skorupa et al., 2012). This process involves syndecans as astrocyte receptors and clathrin-mediated endocytosis as key uptake mechanism. Uptake of angiogenin into astrocytes subsequently modifies the RNA profile of astroglia (Skorupa et al., 2012). Furthermore, uptake of angiogenin into astrocyte was shown to be required for the protection of angiogenin from stress-induced motoneuron injury (Skorupa et al., 2012). An attractive hypothesis derived from these studies is that angiogenin may represent a “help me” signal secreted from stressed motoneurons that stimulates defense mechanisms in astrocytes (Figure 1B). Likewise, it is possible that secreted angiogenin may act on endothelial cells to promote angiogenesis, thereby increasing blood supply to “stressed” motoneurons (Figure 1B).

## RNA Metabolism in ALS and Other Neurodegenerative Diseases

RNA cleavage in motoneurons, astrocytes, or other target cells by angiogenin may significantly alter their RNA metabolism. The current knowledge about RNA metabolism in neurons has been comprehensively reviewed by Strong (2010). Neurons present asymmetrical protein translation, i.e., neurons are able to direct a site-specific protein translation by “packaging” and transporting quiescent mRNA through the cell within ribonucleoprotein (RNP) complexes, also known as *RNA granules*. There are three main types of RNA granules in a mature neuron: (a) transport granules, which contain translationally silent RNA; (b) P-bodies or degradative granules, responsible of mRNA decay; and (c) stress granules (SG), which sequester mRNA in a translationally silent state at times of neuronal injury.

**Figure 2:**
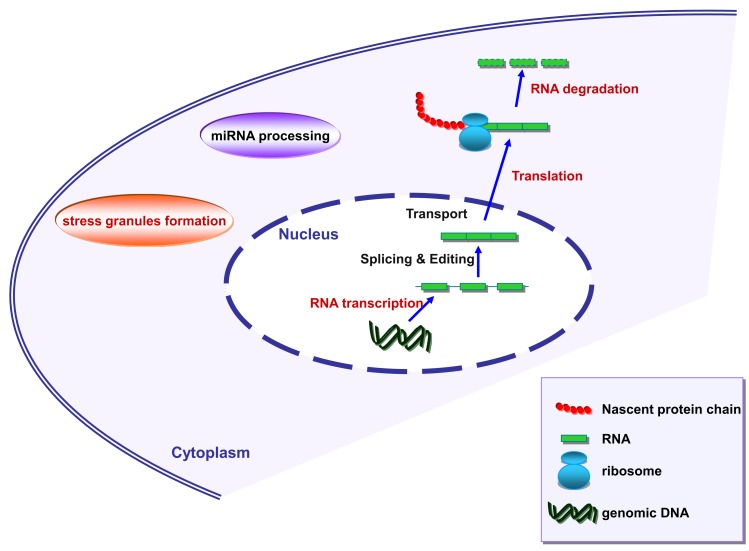
**Schematic representation of RNA metabolism in mammalian cells**. Marked in red are the processes where angiogenin has been shown to be involved.

Stress granules assemble transiently under stressful conditions such as hypoxia, starvation, or exposure to radiation and are able to reprogram RNA translation. Interestingly, angiogenin has also been linked to SG assembly (Emara et al., 2010). Moreover, both TDP-43 and FUS are known to associate with SG (Colombrita et al., 2009; Bosco et al., 2010; Liu-Yesucevitz et al., 2010; reviewed in Dewey et al., 2012). TDP-43 and FUS seem to be implicated in transcription regulation, splicing regulation, miRNA processing, mRNA transport, translation, and decay (reviewed by Lagier-Tourenne et al., 2010; Figure 2). The detailed role of angiogenin in the regulation of these processes therefore warrants further investigations.

Angiogenin is known to stimulate the transcription of rRNA (Li and Hu, 2010) and represents the ribonuclease responsible for the generation of tRNA-derived, stress-induced small RNAs, also known as tiRNAs (Fu et al., 2009; Yamasaki et al., 2009; Ivanov et al., 2011). These tiRNAs are capable of inhibiting protein translation when cells are submitted to stress conditions, such as heat shock, hypothermia, hypoxia, starvation, and radiation. Furthermore, in an elegant study, Emara et al. (2010) demonstrated that angiogenin-generated tiRNAs are able to stimulate the formation of SG. This observation potentially puts angiogenin in interaction with other SG-related proteins, such as TDP-43 and FUS/TLS in the context of ALS.

In our model of angiogenin-mediated neuroprotection, we observed the intraneuronal generation of RNA fragments of consistent size to tiRNAs, however angiogenin internalized by astrocytes generates RNA fragments of different sizes, suggesting that it processes different substrates (Skorupa et al., 2012). RNA processing in astroglia may therefore specifically alter the translational output of astroglia. Two possible mechanisms of action emerge from these observations: (1) angiogenin could *inhibit* the astrocytic production of toxic molecules, or (2) angiogenin would *induce* the astrocytic production of protective molecules. In both hypotheses, angiogenin actions could be mediated by the reprogramming of the protein profile of astrocytes. A third possibility, where angiogenin would be both down-regulating death signals and up-regulating survival signals cannot be ruled out (Figure 1).

Supporting the first scenario, evidence for a pathological role of glia on motoneurons death in ALS has been clearly established, and activated microglia, astrogliosis, and infiltrating lymphocytes coincide with motoneuron injury in ALS spinal cord (Appel et al., 2011). In addition, the toxic effect of astrocytes derived from ALS patients or mouse models on motoneurons has been recently reported (Diaz-Amarilla et al., 2011; Haidet-Phillips et al., 2011). Further studies are therefore required to explore whether angiogenin alters the secretome of astroglia.

## Conclusion

It is not yet clear whether the pathological role of astrocyte and/or other glial cells on ALS disease progression is simply that of increased toxicity, or instead of failure to provide adequate protection against stress signals – internal and/or external. One appealing possibility is a model where an initial stress signal (“hit”) would trigger neurotoxicity. In this scenario, angiogenin (or other ALS-associated proteins) could function as a “rescue message” to astrocytes. Loss-of-function mutations in the *ANG* gene could dramatically increase the susceptibility of motoneurons to stress-induced injury. From a therapeutic perspective, angiogenin delivery may be a viable approach for the treatment of ALS or other neurodegenerative disorders.

Of note, a first “hit” could also be the presence of another ALS-related mutation, such as TDP-43, FUS, or SOD1 mutant proteins (the latter known to exercise its pathological effect through a toxic gain-of-function profile). Cases of ALS patients with mutations in more than one gene have been observed (see Table 1 for reference), as well as the observation of angiogenin mutations, previously linked to ALS, in healthy control subjects (Corrado et al., 2007). This so-called “double hit” hypothesis could be one possible explanation for an ALS scenario where many minor insults or individually harmless genetic polymorphisms put together or acting synergistically, could cause the disease phenotype. Corroborating this hypothesis, a recent study has demonstrated that the frequency of families with multiple mutations is higher than one might expect on the basis of chance (*P* = 1.57 × 10^−7^; van Blitterswijk et al., 2012).

Notwithstanding the cumulative evidence gathered thus far, the role of angiogenin in the physiology and pathophysiology of the nervous system, in particular ALS and Parkinson’s disease, requires further investigation. It is tempting to speculate that the neuroprotective role of angiogenin occurs via a double action both on motoneurons and astrocytes through the reprogramming of protein synthesis. Therefore, the identification of angiogenin substrates and products, together with the understanding of their physiological roles during the context of neurodegeneration may pave the way to new exciting therapeutic possibilities.

## Conflict of Interest Statement

The authors declare that the research was conducted in the absence of any commercial or financial relationships that could be construed as a potential conflict of interest.
